# Supplementary material for: Environmental Impacts of the Brazilian Egg Industry: Life Cycle Assessment of the Battery Cage Production System
Source: Animals (Basel). 2024 Mar 11;14(6):861. doi: 10.3390/ani14060861 (PMC10967559; doi:10.3390/ani14060861)
Supplement: Supplementary file 1 [file animals-14-00861-s001.zip › animals-2878647-supplementary.pdf]

Supplementary Information

Supplementary S1

Table S1: Calculation of nitrogen emissions, methane, and phosphorus losses, considering the functional unit of 1000 hens produced in cages.

**Number of hens**		1025.00
**Excreted N (kg)**		162.06
**Excreted P(kg)**		33.29
**Estimates of nitrogen emissions**		
**Description of emissions**	**Emission factor**	**Emitted N**
Direct emissions of N_2O from manure management ((kg N2O-N (kg N)-1)	0.005	1.27
Volatilization of N as NH_3 and NOx ((kg NH3-N + NOx-N) (kg N)-1))	0.21	34.03
Indirect emissions of N_2O due to NH_3/NOX volatilization ((kg N2O-N) (kg NH3-N + NOx-N)-1))	0.01	0.53
Leaching of NO_3 (kg N (kg N)-1)	0.24	38.89
Indirect emissions of N_2O due to leaching (kg N2O-N (kg N)-1)	0.011	0.67
**Description of emissions**	**Emission factor**	**Emitted N**
Direct emissions of N_2O after application to the soil ((kg N2O-N (kg N)-1)	0.01	1.61
Volatilization of N as NH_3 and NOx after application to the soil ((kg NH3-N + NOx-N) (kg N)-1))	0.21	26.89
Indirect emissions of N_2O due to NH_3/NOX volatilization ((kg N2O-N) (kg NH3-N + NOx-N)-1))	0.01	0.34
Leaching of NO_3 in the soil (kg N (kg N)-1)	0.24	29.56
Indirect emissions of N_2O due to leaching (kg N2O-N (kg N)-1)	0.011	0.43
Total N2O (kg)		4.85
Total NO3 (kg)		68.45
Total NH3 (kg)		48.92
Total NOx (kg)		12.00
**Estimates of methane emissions**		
**Emissions description**	**Emission factor**	**Emitted CH4**
CH4 (kg)	10.5	27.01
**Estimates of phosphorus losses**		
**Emissions description**	**Emission factor**	**Emitted P**
P2O5 (kg)	0.03485	3.56

Table S2. Calculation of nitrogen emissions, methane, and phosphorus losses, considering the functional unit of one ton of eggs produced.

**Number of hens**		55.73
**Excreted N (kg)**		57.80
**Lost P (kg)**		11.36
**Estimates of nitrogen emissions**		
**Emissions description**	**Emission factor**	**Emitted N**
Direct emissions of N_2O from manure management ((kg N2O-N (kg N)-1)	0.005	0.45
Volatilization of N as NH_3 and NOx ((kg NH3-N + NOx-N) (kg N)-1))	0.21	12.14
Indirect emissions of N_2O due to NH_3/NOX volatilization ((kg N2O-N) (kg NH3-N + NOx-N)-1))	0.01	0.19
Leaching of NO_3 (kg N (kg N)-1)	0.24	13.87
Indirect emissions of N_2O due to leaching (kg N2O-N (kg N)-1)	0.011	0.24
**Emissions description**	**Emission factor**	**Emitted N**
Direct emissions of N_2O after soil application ((kg N2O-N (kg N)-1)	0.01	0.75
Volatilization of N as NH_3 and NOx after soil application ((kg NH3-N + NOx-N) (kg N)-1))	0.21	15.78
Indirect emissions of N_2O due to NH_3/NOX volatilization ((kg N2O-N) (kg NH3-N + NOx-N)-1))	0.01	0.16
Leaching of NO_3 in the soil (kg N (kg N)-1)	0.24	18.03
Indirect N_2O emissions due to leaching (kg N2O-N (kg N)-1)	0.011	0.20
Total N2O (kg)		1.99
Total NO3 (kg)		31.91
Total NH3 (kg)		22.42
Total NOx (kg)		5.50
**Estimates of methane emissions**		
**Emissions description**	**Emissions factor**	**Emitted CH4**
CH4 (kg)	10.5	3.18
**Estimates of phosphorus emissions**		
**Emissions description**	**Emission factor**	**Emitted P**
P2O5 (kg)	0.03485	1.21

Supplementary S2

Table S3: Compositions of the feeds for the Pullets houses

Ingredients	Pre-starter Feed	Starter Feed	Grower Feed	Maturity Feed	Pre-laying Feed
Maize	648.062	661.132	661.912	647.677	645.059
Soybean meal	240.849	206.528	196.788	176.726	194.710
Soybean hull	45.405	65.336	77.144	115.491	59.131
Limestone	6.205	9.063	12.954	12.333	51.167
Meat and Bone Meal	42.447	41.686	37.956	38.680	39.876
Methionine	3.987	3.711	2.837	1.257	2.502
Salt	3.500	3.500	3.500	3.500	3.500
Mineral and vitamin	3.000	3.000	3.000	3.000	2.000
Lysine	2.955	2.642	1.544		0.343
Threonine	1.376	1.117	0.961	0.036	0.223
Vitamins D	1.000	1.000	0.250	0.250	0.250
Enzymes	0.500	0.500	0.500	0.500	0.500
Organic acid	0.400	0.400	0.400	0.400	0.400
Tryptophan	0.214	0.285	0.104		0.239
Probiotic	0.100	0.100	0.150	0.150	0.100

Total quantity of 14,435.11 tons of feed produced for pullets in 2021.

Table S4: Compositions of the feeds for the Laying houses

Ingredients	Starter Feed	Peak Feed	Laying Feed 1	Laying Feed 2	Laying Feed 3
Maize	602.221	622.461	626.634	633.372	624.566
Soybean meal	174.642	151.460	147.150	136.581	133.616
Limestone	93.156	94.523	102.652	108.835	106.486
DDG	80.000	80.000	80.000	80.000	80.000
Meat and Bone Meal	32.044	27.502	17.564	14.497	11.355
Soybean hull	-	9.947	15.023	15.601	24.484
Seaweed	-	-	-	-	10.000
Vegetable oil	5.349				
Methionine	2.676	2.747	1.665	1.460	1.073
Sodium Sulfate	2.000	2.500	2.500	2.500	2.500
Salt	2.000	2.000	2.000	2.000	2.000
Mineral and vitamin	2.000	2.000	2.000	2.000	2.000
Lysine	1.278	1.906	0.616	0.664	0.476
Mycotoxin Adsorbent	1.000	1.000	1.000	1.000	
Threonine	0.460	0.688	-	-	-
Choline chloride	0.500	0.500	0.500	0.500	0.500
Enzyme Bland	0.500	0.500	0.500	0.500	0.500
Tryptophan	0.174	0.266	0.196	0.240	0.194
Vitamin D	-	-	-	0.250	0.250

Total quantity of 157,267.89 tons of feed produced for laying hens in 2021.

Supplementary S3

Table S5: Data quality criteria and rating system: ecoinvent pedigree matrix.

	1	2	3	4	5
**Reliability**	Verified data based on measurements	Verified data partly based on assumptions or non-verified data based on measurements	Non-verified data partly based on qualified estimates	Qualified estimates (e.g. by industrial expert)	Non-qualified estimates
**Completeness**	Representative data from all sites relevant for the market considered, over and adequate period to even out normal fluctuations	Representative data from >50% of the sites relevant for the market considered, over an adequate period to even out normal fluctuations	Representative data from only some sites (<50%) relevant for the market considered, or >50% of sites but from shorter periods	Representative data from only one sites relevant for the market considered, or some sites but from shorter periods	Representativeness unknown or data from a small number os sites and from shorter periods
**Temporal correlation**	Less than 3 years of difference to the time period of the data set	Less than 6 years of difference to the time period of the data set	Less than 10 years of difference to the time period of the data set	Less than 15 years of difference to the time period of the data set	Age of data unknown or more than 15 years of difference to the time period of the data set
**Geographical correlation**	Data from area under study	Average data from larger area in which the area under study is included	Data from area with similar production conditions	Data from area with similar production conditions	Data from unknown or distinctly different area (North America instead of Middle East, OECD-Europe instead of Russia)
**Further technological correlation**	Data from enterprises processes and materials under study	Data from processes and materials under study (i.e. identical technology) but from different enterprises	Data from processes and materials under study but from different technology	Data on related processes and materials	Data on related processes on laboratory scale or from different technology

Source: Ciroth et al. [1].

Supplementary S4

Table S6: Data quality assessment: ecoinvent pedigree matrix.

Life cycle stage	Data	R	C	T	G	F
Feed Production	Feed composition	1	1	1	1	1
	Direct energy use	1	1	1	1	1
	Material and energy inputs	1	2	1	2	2
Pullet Facilities	Feed Composition	1	1	1	1	1
	Material and energy Inputs	1	1	1	1	1
	Manure management	2	3	1	2	1
	Mortalities	2	2	1	2	1
Layer Facilities	Feed Composition	1	1	1	1	1
	Material and energy Inputs	1	1	1	1	1
	Manure management	2	3	1	2	1
	Mortalities	2	2	1	2	1
Washing & Grading	Material and energy inputs	2	2	1	2	1
Egg Breaking and Further Processing	Material and energy inputs	2	2	1	2	2
Transportation	Transportation distances (foreground data)	1	1	1	1	1

The abbreviations for the columns are **R**eliability, **C**ompleteness, **T**emporal correlation, **G**eographical correlation, **F**urther technical correlation as defined in the data quality scheme, according to Ciroth et al. [1].

REFERENCE

1. Ciroth, A.; Noi, C.; Lohse, T.; Srocka, M. OpenLCA 1.10 - Comprehensive User Manual. 2020, 127. Available online: https://www.openlca.org/wp-content/uploads/2020/01/openLCA_1.10_User-Manual.pdf. (accessed on 1 March 2023)
